# Heterocellular cadherin connections: coordinating adhesive cues in homeostasis and cancer

**DOI:** 10.12688/f1000research.11357.1

**Published:** 2017-06-27

**Authors:** Silvia Fontenete, Daniel Peña-Jimenez, Mirna Perez-Moreno

**Affiliations:** 1 Epithelial Cell Biology Group, Cancer Cell Biology Programme, Spanish Cancer Research Centre (CNIO), Madrid, Spain

**Keywords:** Cadherin catenin, heterocellular adhesion, heterotypic adhesion, cancer, Cadherin adhesion

## Abstract

This short insight covers some of the recent topics relevant to the field of cadherin–catenin adhesion in mediating connections between different cell types, so-called heterotypic or heterocellular connections, in both homeostasis and cancer. These scientific discoveries are increasing our understanding of how multiple cells residing in complex tissues can be instructed by cadherin adhesion receptors to regulate tissue architecture and function and how these cadherin-mediated heterocellular connections spur tumor growth and the acquisition of malignant characteristics in tumor cells. Overall, the findings that have emerged over the past few years are elucidating the complexity of the functional roles of the cadherin–catenin complexes. Future exciting research lies ahead in order to understand the physical basis of these heterotypic interactions and their influence on the behavior of heterogeneous cellular populations as well as their roles in mediating phenotypic and genetic changes as cells evolve through complex environments during morphogenesis and cancer.

## Introduction

Cadherin–catenin-mediated adhesion at adherens junctions (AJs) is fundamental for the establishment of the physical association between cells in multicellular organisms, coordinating the arranged and polarized development, architecture, and function of tissues ^1– 3^. The last several years of scientific discovery have been instrumental in understanding the dynamic structure and regulation of the stability of the cadherin–catenin complexes at the membrane as well as the connection of these complexes with the cytoskeleton. In addition, these findings also unveiled roles for cadherin complexes beyond their structural function such as directing cell polarity or behaving as sensors of mechanical inputs and signaling cues. Each of these cadherin–catenin functions features prominently in the regulation of several aspects of cell behavior, including cell proliferation, cell fate, and cell migration during development and homeostasis, and their importance is confirmed when these functions go awry in disease. Many of these findings have been thoroughly documented by several excellent reviews elsewhere ^1– 11^. In this short review, we highlight some advances in the role of mammalian classical cadherins that have emerged in the past decade beyond their function of mediating homotypic adhesion (between equal cell types) and focus on their part in coordinating cell behavior by establishing heterotypic or heterocellular connections (between different cell types) in homeostasis and cancer.

## Organization of the cadherin–catenin complex

**Figure 1.**
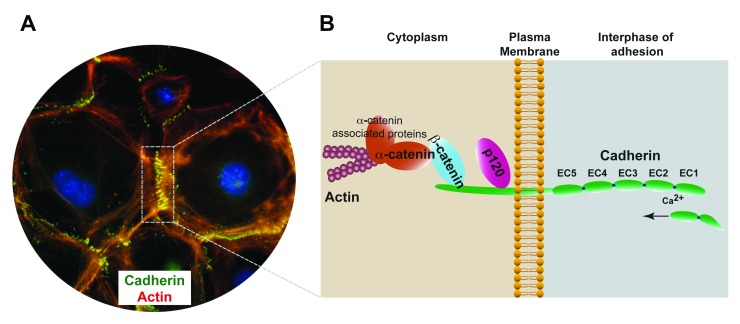
Structural organization of classical cadherin–catenin complexes. **A**) Epithelial cells establishing adhesive cadherin interactions (E-cadherin, green; actin, red). **B**) Schematic organization of the cadherin–catenin complexes. Cadherin transmembrane adhesion receptors establish dynamic adhesive interactions within opposing membranes in neighboring cells. Through the cadherin intracellular domain, cadherin molecules interact with p120-catenin (p120) and β-catenin. p120 binds directly to the juxtamembrane domain of the cadherin tail and β-catenin binds to the cadherin C-terminal domain, mediating the connection with α-catenin, which in turn mediates the association of the cadherin complex to the actin cytoskeleton. EC1–5, extracellular cadherin subdomain 1–5.

Cadherins belong to a superfamily of proteins defined by a shared ectodomain that presents a tandem of an immunoglobulin-like module defined as the extracellular cadherin (EC) repeats ^8, 12, 13^. Based on this structure, cadherins can be classified into several subfamilies ^14^. Here, we focus our attention on the classical subfamily of cadherins, since their function in mediating adhesive interactions at AJs between adjoining cells has been better defined. The paradigmatic organization of classic cadherin junctional complexes involves the presence of a single pass cadherin transmembrane adhesion receptor presenting five extracellular calcium-binding EC repeats. This extracellular domain establishes dynamic adhesive interactions with opposing membrane-embedded cadherin complexes in neighboring cells ^1– 3^. The stabilization of these adhesive contacts occurs via the intracellular domain of the cadherin molecule through interaction with the catenin proteins p120-catenin (p120) and β-catenin, which dynamically regulate cell adhesion as well as other aspects of cell behavior ^1– 3^. p120 binds directly to the juxtamembrane domain (JMD) of the cadherin tail and controls its stability at the plasma membrane. β-catenin, although well known for its signaling function in the Wnt pathway, also binds the C-terminal domain of cadherins through the catenin-binding domain, mediating the connection with α-catenin. In turn, α-catenin interacts with actin-binding proteins connecting the cadherin complex to the actin cytoskeleton ^3, 4^ ( Figure 1).

## Establishing classical cadherin connections

The different members of the classical cadherin subfamily were initially named after the tissue in which they are preferentially expressed (e.g. epithelial [E], neural [N], placental [P], retinal [R], and vascular endothelial [VE]). These adhesion receptors are mainly involved in homophilic cell–cell interactions (between identical cadherins); however, heterophilic cadherin cell–cell interactions (between different cadherins) can also occur, as we will discuss later in the text. Upon ligation, the EC repeats strengthen the adhesive binding by mediating not only trans interactions between cadherins on opposing membranes but also cis interactions, leading to the formation of cadherin dimers, oligomers, and clusters. This modular organization at the interphase of adhesion of adjacent cells provides adhesive cells mechanical resistance and strength, allowing them to respond as a coordinated tissue to cues arising from the environment ^3– 6^.

Recent advances have provided novel insight into the mechanisms underlying clustering regulation through key cortical regulators ^15^. In the absence of cadherin ligation, cadherins are able to organize into minimal clusters in a cortical actin-dependent manner. Under these conditions, cortical actin acts as a fence, limiting the dispersion of cadherin complexes ^16, 17^. Upon cadherin ligation, the formation of cadherin clusters increases, coordinating intercellular adhesion ^18^. At the cadherin cytoplasmic domain, the strength of adhesion is regulated by the former’s binding to catenins and by the direct or indirect association of other intracellular molecules that link the cadherin–catenin complex to the actin cytoskeleton ^2^. Among these molecules, the Rho GTPase family members Rho, Rac, and Cdc42 play a critical role. In particular, each molecule performs specific functions in maturation, polarized distribution, or tension promotion via actomyosin contractility, leading to the formation of robust cell–cell interactions ^2^. Importantly, the crosstalk between AJs and Rho GTPases is bidirectional: Rho GTPases participate in the formation and maintenance of AJs, whereas AJs modify the activity of these GTPases, leading to changes in the structure and polarity of the cell ^11^. Owing to their relevance in development and disease, substantial efforts have been made to understand the mechanisms that regulate the expression, functional activity, and binding specificity of cadherins and thus the adhesive properties of cells.

## Cadherin binding specificity and cell sorting

The expression of specific members of the cadherin subfamily has a major role in allocating defined cell types to their proper positions during development, generating defined tridimensional structures that are important for tissue function ^8, 19^. The involvement of differential cadherin binding in cell sorting was initially addressed using cell aggregation assays carried mainly in cell suspensions. In these assays, cells transfected with different cadherin subtypes aggregated only with cells expressing the same cadherin molecule but not with cells expressing other cadherin subtypes ^20– 22^. This indicated that the nature of cadherin-mediated cell adhesion is selective and led to the notion that homophilic cadherin interactions direct the differential distribution of cellular populations.

The cell-sorting phenomenon is observed in several developmental processes, such as the formation of the neural tube in vertebrates, where the differential expression of E- and N-cadherin contributes to the separation of the N-cadherin-positive neural tube cells from the E-cadherin-positive embryonic ectoderm layer. During this process, the ectodermal cells switch their expression of E-cadherin to the expression of N-cadherin through an epithelial-to-mesenchymal transition (EMT) process, where epithelial cells acquire mesenchymal features as well as migratory and invasive characteristics. The expression of N-cadherin confers on neural cells, among other features, a different adhesive property, facilitating their displacement from the ectoderm and the formation of the neural tube, where cells homotypically associate through N-cadherin ^23^. In adult tissues, the relevance of cadherin-mediated adhesion in cell sorting is best exemplified by its role in EMT and metastasis, where the loss of E-cadherin expression, along with the upregulation of N-cadherin, facilitates the displacement of N-cadherin invasive cells from the primary tumor, which is considered a hallmark of malignancy ^24, 25^. The occurrence of this cadherin switch in biological processes fostered further the concept of a role of homophilic and homotypic cadherin interactions in cell sorting during development, tissue repair, and cancer.

However, several lines of evidence have uncovered the existence of adhesive interactions between cells expressing different cadherin molecules ^26– 29^. This was also observed in adhesion experiments of cells bound to immobilized cadherin ectodomains of different cadherin subtypes ^30^. These assessments of cadherin ligation were conducted under shear forces and exposed the relevance of the strength of adhesion rather than the specific expression of a particular cadherin subtype in cadherin-mediated cell sorting. The aforementioned binding of different cadherin subtypes involves both the expression levels of a given cadherin subtype and the strength of adhesion ^28^. Thus, cell segregation is not regulated by cadherin binding specificity but by cadherin-relative levels, affinity, and physical strength, in agreement with the differential adhesion hypothesis postulated by Steinberg ^31^. Although the physical basis of these cadherin interactions is not completely understood, some of the functional effects of cadherin heterotypic interactions on different aspects of cell behavior have emerged in past years, as we discuss in the next section.

## Cadherin binding: connecting different cell types

The development and function of organs involve a highly dynamic and complex coordination of multiple cell types within tissues to maintain their architecture and fulfill their specialized tasks, enabling them to adapt to environmental changes. These interactions start from the formation of the germ layers: the ectoderm, mesoderm, and endoderm ^32^. Early in organogenesis, epithelial and mesenchymal cells derived from different germ layers interact spatiotemporally, giving rise to the diverse body plans that result in functional organs. These developmental processes require complex gene networks, cell signaling, and gene-regulated cell behaviors such as cell division, adhesion, repulsion, polarization, apoptosis, contraction, extracellular matrix secretion, and signal secretion and reception ^33^. The dynamic regulation of cadherin cell adhesion and its specific spatiotemporal expression pattern is critical during development and adult tissue homeostasis, allowing the establishment of connections with similar and different cellular types present in the tissue. For example, in normal skin, E-cadherin homotypic adhesions are established between epidermal keratinocytes and Langerhans cells ^34^. In a similar scenario, keratinocytes are able to establish interactions with melanocytes and Merkel cells via E-cadherin ^35, 36^ and P-cadherin ^36, 37^, allowing the proper distribution and functioning of these different cell types within the tissue. However, several reports have also indicated the existence of heterocellular interactions between different mammalian cell types mediated by different cadherin subtypes.

Some of the earliest observations indicating the existence of N- and E-cadherin heterotypic interactions between mammalian cells were made between liver cells and fibroblasts ^38, 39^, and such interactions induced the reorganization of the actin cytoskeleton into lamella-like structures ^38^. This phenomenon was also observed in co-cultures of liver and retinal cells expressing N-cadherin and L-CAM, respectively ^26^, and between N-cadherin-expressing fibroblasts and E-cadherin-expressing MDCK cells, but since the latter also express other cadherin subtypes, this might have contributed to establishing adhesive interactions ^38^. Although concomitant expression of E- and N-cadherin was not found in epithelial cells in any of the cellular systems mentioned above, both E- and N-cadherin were found to be expressed in hepatocytes and liver carcinoma cells forming adhesive structures between neighboring cells and fibroblasts ^40^. Which, then, are the molecular events that dictate the establishment of homotypic or heterotypic cadherin interactions that may account for the selective association of different cell types? The physical basis for these interactions is still not completely understood, but some findings have provided insightful information regarding the differential dimerization affinities between homophilic and heterophilic cadherin interactions. In this regard, laminar flow approaches, biophysical studies, and structural analyses have shown that N-cadherin forms homodimers with higher affinity than those formed by E-cadherin. But, when these two cadherins form trans-heterophilic dimers, the strength of their binding affinities is higher than the E-cadherin homophilic bonds ^30, 41, 42^. This process could also be potentially determined by the cadherin levels present at the cell membrane that are available to establish these interactions ^7, 9, 10^ and by mechanical forces exerted through the association of the cadherin molecules at the cytoplasm with the actin cytoskeleton ^4– 6^. Thus, the selective association into homophilic or heterophilic cadherin interactions may be attributable in part to a combination of differential affinities between the cadherin bonds, the surface levels of each of these molecules in different cell types, and the adhesive strength and mechanical forces. These findings suggest that a balanced ratio between homophilic and heterophilic cadherin interactions facilitates the existence of both types of interactions between the different cell types that dwell in specific tissues. However, when this balance is impaired and favors stronger heterotypic interactions, these may lead to changes in tissue patterning as well as increased associations between different cell types. Different heterocellular interactions have been documented. Here, we focus on those established by epithelial cells and fibroblasts, since they have started to be better defined.

Fibroblasts are the most abundant cell type found in the stroma surrounding epithelial tissues. In cancer, fibroblasts can promote tumor growth and the acquisition of malignant characteristics by secreting tumor-promoting factors ^43^. A recent study has provided relevant insight into how the direct contact of fibroblasts with invading human carcinoma cells controls their invasive characteristics ^44^. In this study, cancer cells were found to associate directly with fibroblasts by establishing E- and N-cadherin heterotypic interactions, which attained adhesive resistance and mechanical strength, allowing the collective invasion of cancer cells through their associations with α-catenin/vinculin. Interestingly, the fibroblasts that were directionally attracted and migrating towards cancer cells, upon heterotypic binding, inverted their front/rear polarity in an N-cadherin- and afadin-dependent manner and exerted pulling forces onto tumor cells fostering their collective invasion ^44^. Compounding the underlying complexities associated with tumorigenesis, the functional consequences of the establishment of heterotypic cadherin interactions between cancer cells and fibroblasts in promoting collective invasion are opening up a new way in which tumors exploit the tumor-promoting microenvironment to acquire malignant characteristics, in particular in metastatic tumors that do not undergo EMT events and still maintain the expression of E-cadherin at the membrane ^45, 46^. Interestingly, the metastatic potential of E-cadherin-expressing tumor cells has also been associated with reductions in the functional activity of E-cadherin to mediate adhesive homotypic interactions ^47^. Putting both scenarios together, an interesting possibility is that, under these conditions, stronger heterotypic cadherin adhesions may be rather favored, allowing the interactions of cancer cells with tumor-promoting stromal cells. Whether other cellular types or tissue-specific differences may render different cellular responses upon the establishment of heterocellular cadherin adhesion is still an open question for the future. In this regard, interactions between melanoma cells with fibroblasts via N-cadherin have been shown to be required for their survival, while the interaction of melanoma cells with endothelial cells by N-cadherin has been involved in their transendothelial migration ^48, 49^.

In addition to fibroblasts, the tissue microenvironment comprises numerous cell types that have a major role in the regulation of tissue homeostasis and the outcome of tumor malignancy. One characteristic of developing tumors is the presence of inflammatory microenvironments that may exert an inhibitory or a promoting effect on tumorigenesis ^50– 53^. In recent years, several findings have unveiled the expression of cadherins in immune cells including T cells, dendritic cells, Langerhans cells, and macrophages and the adhesive interactions mediating the connection between immune cells or between immune cells and epithelial or endothelial cells. Although the physical basis of these associations is not completely understood, some of their roles in modulating immune function have surfaced in the past few years ^54– 57^. In normal skin, keratinocytes and Langerhans cells associate through homotypic E-cadherin adhesion ^34^, regulating Langerhans cell retention as well as holding them in an undifferentiated state ^58^. In the intestinal epithelia, epithelial cells establish heterotypic interactions with T lymphocytes ^59^. This can be mediated through the binding of T cells expressing αEβ7integrin (also known as CD103) with E-cadherin in epithelial cells ^60^. αEβ7integrin-positive T cells provide surveillance against harmful infections, transform epithelial cells, and participate in tissue repair. One example is the cytolysis of pancreatic carcinoma cells that maintain the expression of E-cadherin at the membrane through the heterotypic interactions with αEβ7integrin-positive T cells ^61^. Other specific subsets of T lymphocytes express another receptor for E-, N-, and R-cadherins known as killer cell lectin-like receptor G1 (KLRG1) ^62– 64^, which is an inhibitory receptor expressed on steady-state natural killer (NK) cells as well as in CD8+ T cells. Through these heterotypic interactions, E-cadherin is able to regulate TCR signaling, while KLRG1 in turn regulates cell adhesion dynamics and E-cadherin signaling in epithelial cells ^55^. Additional studies have also shown that E-cadherin-mediated adhesion regulates the functions of innate immune cells, such as mononuclear phagocytes. E-cadherin is expressed by alternatively activated M2 macrophages ^65, 66^, which are tightly associated with fostering tumor-promoting microenvironments ^67^. Interestingly, the expression of E-cadherin is not necessary for M2 polarization *in vivo* ^68^, but it allows the heterotypic association of macrophages with T cells expressing CD103 and KLRG1, potentially regulating their retention in tissues and their polarization ^66^. Future research may increase our understanding of the potential implications of these emerging heterocellular associations mediated by cadherins in modulating the function of immune cells in the context of cancer and tumor plasticity.

## Future directions

It is well established that cadherin-mediated adhesion is an important determinant for development, tissue architecture, and function and that the loss or alterations in the functional activity of cadherins are important determinants for tumor progression. In this short overview, we summarized some recent findings that are increasing our awareness of the numerous heterocellular interactions established by mammalian classical cadherins. Future research will lead to a better understanding of the growing complexity of these connections. For example, what is the physical basis of the establishment of these heterotypic interactions, and what dictates the biological differential affinities between different cell types? With the current technological advances at hand, including super resolution imaging as well as structural, engineering, and biochemical approaches, future research will soon shed light onto the molecular nature of these heterotypic interactions in defined cellular populations and the involvement of the strength of adhesion in these events. In addition, live cell imaging and mechanical and ultrastructural analyses of cells establishing heterotypic interactions within tissues will be instrumental to the elucidation of the role of these connections in morphogenesis, homeostasis, and cancer. The particular features of these connections regulating cell adhesion dynamics, mechanics, and signaling may underlie additional levels of control of different cellular processes, including cell sorting, cell polarity, and cell division, and overall tissue organization or immune regulation, enabling cells to sense, signal, and respond to specific spatiotemporal changes in their environment. Moreover, tumors have now come to be understood to function as complex tissues in which numerous cells, collectively termed the tumor microenvironment, play a critical role. Thus, elucidating the role of heterotypic cadherin interactions in regulating chronic inflammation, cell growth, and survival and the malignant characteristics of clonal populations of tumor cells in specific tumors will reveal additional mechanisms of tumorigenesis as cells evolve through complex environments that may spawn a new era of therapeutic strategies directed towards the eradication of tumors.

## Abbreviations

AJ, adherens junction; EC, extracellular cadherin; EMT, epithelial-to-mesenchymal transition; JMD, juxtamembrane domain; KLRG1, killer cell lectin-like receptor G1; NK, natural killer; p120, p120-catenin.
